# Social Media Use and Vulnerable Narcissism: The Differential Roles of Oversensitivity and Egocentricity

**DOI:** 10.3390/ijerph18179172

**Published:** 2021-08-31

**Authors:** Rebecca B. Fegan, Amy R. Bland

**Affiliations:** Department of Psychology, Faculty of Health and Education, Manchester Metropolitan University, Manchester M15 6GX, UK; REBECCA.FEGAN@stu.mmu.ac.uk

**Keywords:** vulnerable narcissism, grandiose narcissism, oversensitivity, egocentricity, social media

## Abstract

Narcissism is a multi-dimensional personality trait characterised by grandiosity, vanity, low empathy, and a desire for admiration. Previous research has suggested that social media sites are ideal platforms for people with narcissistic traits to satisfy a desire for attention. However, less is understood about the relationship between social media and vulnerable narcissism, characterised by vulnerability, insecurity, and oversensitivity. A total of 115 participants completed the Hypersensitive Narcissism Scale (HSNS) and questions relating to social media use. Exploratory and confirmatory factor analysis supported a two-factor model of vulnerable narcissism; *oversensitivity* and *egocentrism*. Results showed that greater oversensitivity was significantly associated with greater scores in all six aspects of social media use. Specifically, time spent on social media, frequency of posts, concerns about numbers of likes and comments, being overly sensitive about negative remarks, feelings of jealousy, and a greater difference in how they portray themselves on social media compared to real life. Egocentricity was significantly associated with less concern over negative remarks and a greater difference in how they portray themselves on social media compared to real life. These results suggest that vulnerable narcissism is not a unitary trait and that subfactors *oversensitivity* and *egocentricity* contribute differentially to aspects of social media use.

## 1. Introduction

Narcissism refers to a multi-dimensional personality trait characterised by grandiose fantasies of success and power, self-absorption, selfishness, lack of empathy, an inflated sense of self-importance, entitlement, and a deep need for attention and admiration [1,2]. Narcissism is thought to exist on a spectrum with individuals on the lower end of the narcissism spectrum possessing few narcissistic traits, which may be advantageous given that it encompasses positive traits such as self-confidence, ambition, assertiveness, and resilience [3,4,5]. These traits often offset low-self-esteem and are helpful in achieving leadership positions within competitive fields such as law and business [6]. Alternatively, individuals on the higher end of the narcissism spectrum exhibit more extreme and pervasive patterns of narcissistic traits, often making it difficult for these individuals to maintain romantic relationships and form genuine friendships [7].

Trait narcissism has generally been considered a unitary concept; however, there is growing evidence to suggest that there are at least two forms of trait narcissism, namely grandiose narcissism and vulnerable narcissism [8,9]. Grandiose narcissism, often referred to as “textbook” narcissism, is the most well-known form of narcissism and is characterised by high self-esteem, extraversion, confidence, and social boldness [8,10]. Alternatively, vulnerable narcissism, also referred to as hypersensitive, covert, fragile, or implicit narcissism, is in stark contrast to the self-assuredness of grandiose narcissism. Vulnerable narcissism is characterised by hypersensitivity to the opinions of others, an intense desire for approval, defensiveness, low self-esteem, introversion, neuroticism, and insecurity [8,11,12,13]. Consequently, driven by fear of receiving criticism, vulnerable narcissists’ self-image is tied to external feedback [14] and these individuals tend to engage in fewer social interactions compared with grandiose narcissists [5,8,9,15]. Vulnerable narcissists are thought to be defensive and avoidant and are often unable to promote their perceived level of importance and superiority, and therefore, they do not receive the sufficient amount of admiration and attention that they crave [16,17,18]. This discrepancy between the deep-seated need for attention, and the lack thereof, violates an already fragile self-esteem, leading to increased feelings of anxiety and depression and in turn encourages further avoidance, defensiveness, and social withdrawal [8,19,20]. Ultimately, grandiose narcissism has been linked with increased life satisfaction and happiness, whereas vulnerable narcissism has been linked with feelings of depression, anxiety, and dissatisfaction [8].

### 1.1. Narcissism and Social Media Use

Over the last decade, social media use has become an increasingly popular leisure activity across the world [21]. Social media platforms such as Facebook, Twitter, Instagram, Snapchat, and TikTok are both entertaining and beneficial by allowing users to stay in touch with family and friends, share images and content, form professional connections, fundraise for important causes, and engage in discussions with other people on a wide range of topics [22]. The desire for attention and admiration, inherent in trait narcissism, has led researchers to hypothesise that social networking sites are ideal platforms for narcissists to achieve narcissistic goals [23]. A recent meta-analysis of social media and narcissism found that individuals who score high in grandiose narcissism tend to use social media more frequently, post more selfies and status updates, and have more online friends as opposed to those who scored lower in grandiose narcissism [13]. In addition, individuals who scored higher in grandiose narcissism tend to be more susceptible to social media addiction compared with individuals who score lower in grandiose narcissism [23].

The positive association between grandiose narcissism and elevated social media use is thought to stem from the gratification that comes from receiving “likes” and positive comments on pictures and status updates [13,23]. Receiving likes and comments is thought to feed into vanity and desire for attention inherent in grandiose narcissism and reaffirms a sense of self-importance [23,24]. Additionally, the high level of self-control that social media allows users over their self-image enables users to selectively pick and choose what they share online and omit any undesirable images or news about themselves [3]. This allows people who display high levels of grandiose narcissism to curate and refine their “perfect” self-image even further, which ultimately results in an online “self” that only shows what they want to be seen, and garners positive attention even if that “self” is at odds with the narcissists’ reality [23]. Indeed, the Social Online Self-regulation Theory (SOS-T) suggests that social media is an ideal platform for self-regulation, serving as a means to attain a variety of goals, including self-esteem, social interaction, materialistic goals, and self-presentation [25].

To date, research exploring the relationships between social media use and trait narcissism has focused on grandiose narcissism, while the link between social media use and vulnerable narcissism is less well known. It has been suggested that vulnerable narcissism may in fact contribute more to problematic use of social networking sites (SNS) than grandiose narcissism [18]. Indeed, people who are high in vulnerable narcissism feel anxious in face-to-face interactions and feel more comfortable online, which may lead to problematic social media use [26]. A recent meta-analysis by Casale and Banchi (2020) found that studies that have investigated vulnerable narcissism and problematic social media use reported a significant moderate positive association [26,27,28]. Recent studies also suggested that vulnerable narcissism, but not grandiose narcissism, is the primary determinant of Facebook use [29,30]. Furthermore, a study comparing vulnerable and grandiose narcissists found significantly higher levels of problematic internet use and a stronger preference for online social interactions compared to grandiose narcissists [18]. However, another recent meta-analysis, which did not explicitly investigate problematic social media use, found that vulnerable narcissism was not significantly related to (a) time spent on social media, (b) frequency of status updates/tweets on social media, (c) number of friends/followers on social media, and (d) frequency of posting pictures of self or selfies on social media [13]. These somewhat inconsistent findings highlight the need for further research into vulnerable narcissism and the factors which may drive the variance in social media usage. Therefore, this study sought to evaluate the relationship between vulnerable narcissism and frequency of viewing and posting to social media. Furthermore, we were specifically interested in the relationship between vulnerable narcissism and social media usage, which may give rise to negative affect and have consequences for psychosocial development and well-being. This includes concerns over the number of likes and comments [31], the need for social comparison and jealousy of others’ social media posts [32,33], and feeling dejected from negative comments [34]. Finally, as authentic self-expression on social media is associated with greater subjective well-being [35], we sought to evaluate the potential role of vulnerable narcissism in portraying oneself differently on social media [36].

### 1.2. Oversensitivity and Egocentricity

Hendin and Cheek’s [37] Hypersensitive Narcissism Scale (HSNS) was developed to measure vulnerable narcissism. Initially, it was believed that all ten items on the HSNS load onto a single, vulnerable narcissism factor. However, more recent research has re-examined the factor structure of the HSNS, finding that a two-factor structure is more appropriate to account for the variance in reported scores [38,39]. These two factors encompass *oversensitivity*—relating to hypersensitivity to judgment and rejection—and *egocentricity—*relating to self-absorption and self-centeredness. Although there has been some variation as to which items of the HSNS load onto, a recent large-scale study using over 21,000 participants found strong evidence for a two-factor model [38]. The authors define *oversensitivity* as an anxious and vulnerable state with a strong reaction to others perceiving oneself as negative or unfavourable and *egocentricity* as egocentrically overly focused on one’s own needs and desires at the expense and disregard for others’ needs and desires [38]. It has, therefore, been suggested that measures of vulnerable narcissism, such as the HSNS, may also be measuring aspects of overt (i.e., grandiose) narcissism and that the oversensitivity factor may be a purer measure of vulnerable narcissism [38].

The aim of the present study was to identify whether a one-factor or two-factor model would better explain the variation in vulnerable narcissism scores and whether these map onto the items loadings that have been more recently observed. We hypothesised that a two-factor model would relate to *oversensitivity* and *egocentricity*. We further sought to investigate which aspects of social media usage are related to *oversensitivity* and *egocentricity* scores*, s*pecifically, concerns about numbers of likes and comments, being overly sensitive about negative remarks to social media posts, feelings of jealousy about other peoples’ social media posts, and a greater difference in how they portray themselves on social media compared to real life. Given that previous literature is mixed as to whether people higher in vulnerable narcissism use social media more, we also sought to investigate whether higher scores of *oversensitivity* and *egocentricity* would be associated with more time spent on social media and a greater frequency of social media posts.

## 2. Materials and Methods

### 2.1. Participants

In total, 115 participants completed an online survey of their social media use via Qualtrics. Participants were predominantly recruited from Manchester Metropolitan University (MMU) and the Greater Manchester area. Participants’ ages ranged from 18 to 50 years old with a mean age of 23.17 years (*S.D* 6.55), including 89 females, 24 males and 2 individuals who answered “prefer not to say”. Participants completed the survey remotely on either a phone or computer and were allowed an unlimited amount of time to complete the study. There was no financial compensation for completing this study. The research ethics committee at Manchester Metropolitan University approved this study (ref: 22244). All procedures complied with the ethical standards of the relevant national and institutional committees on human experimentation and with the Helsinki Declaration of 1975, as revised in 2008.

### 2.2. Measures

#### 2.2.1. Hypersensitive Narcissism Scale (HSNS)

The Hypersensitive Narcissism Scale (HSNS), developed by Hendin and Cheek [37], is a self-report scale which measures vulnerable narcissism. This scale includes 10 statements which reflect typical vulnerable narcissistic traits. Participants are asked to use a five-point Likert scale to indicate to what extent each statement is characteristic of their own feelings and behaviours; 1 = very uncharacteristic or untrue, strongly disagree, 2 = uncharacteristic, 3 = neutral, 4 = characteristic, 5 = very characteristic or true, strongly agree. Participants were given a total score out of 50. The reported reliability of the HSNS is good, with alpha coefficients ranging from 0.72–0.76 [37]. Furthermore, the HSNS has shown good validity and has been reported to provide the best match to expert ratings of vulnerable narcissism [40].

#### 2.2.2. Social Media

Participants were asked “On average, how much time do you spend looking at social media each day” with a five-point Likert scale, including: “less than 1 h”, “1–2 h”, “2–5 h”, “5–10 h”, and “more than 10 h”. Participants were also asked “How often do you post to your social media accounts?” on a six-point Likert scale with responses including: “never”, “every few months”, “every few weeks”, “a few times a week”, “once a day”, and “multiple times a day”. Furthermore, participants were asked to rate the following statements using a five-point Likert scale (with a score of 1 being “strongly agree” and a score of 5 being “strongly disagree”): “I care about the number of likes and/or comments I receive on the things I post, and I feel good about myself when this number is high”, “I feel upset and/or jealous when I see other people posting positive updates about their life and I find myself comparing my life to theirs”, “One negative remark on my post would make me feel badly and would ruin my day”, and “How I portray myself on social media is different to how I am in real life”. The participants’ relationship with social media was further assessed by asking: “What social media platforms do you use the most?”, “What would you say are your main reasons for using social media?”, and “At what time of the day do you usually check social media?”.

### 2.3. Statistical Analysis

Statistical analyses were conducted in SPSS 25 and JASP (JASP Team (2020), version 0.14.1). The statistical significance level was set to *p* < 0.05 (two-tailed). Effect sizes were estimated using Cohen’s *f*^2^, which allows an evaluation of effect size in a multivariate regression model. According to Cohen’s [41] (1988) guidelines, *f*^2^ ≥ 0.02, *f*^2^ ≥ 0.15, and *f*^2^ ≥ 0.35 represent small, medium, and large effect sizes, respectively. Partial eta squared (η_p_^2^) effect sizes were calculated for ANOVAs. A test of excessive significance (TES [42]) was calculated to obtain the success rate, median observed power, the inflation rate, and the replicability index based on the multiple regression analysis. For these calculations we used the p-checker-app (see http://shinyapps.org/apps/p-checker/ (accessed on 12 August 2021)).

#### 2.3.1. Factor Analysis

We entered all 10 items of the HSNS into an exploratory factor analysis to determine the underlying latent variable structure of the data. Only eigenvalues greater than 1 were used to determine whether a factor explained sufficient variability in the data. The method employed utilised varimax rotation with Kaiser normalisation.

#### 2.3.2. Hierarchical Regression

A series of six hierarchical regression analyses were conducted to investigate whether the variables *oversensitivity* and *egocentricity* are related to aspects of social media use. Assumptions of normality, independence, and multicollinearity of the residues were verified. In addition, the independence of the residues was verified through the Durbin–Watson statistic (all = 1.60–2.36), this value being within the suggested threshold [43]. Multicollinearity was tested through tolerance (0.94) and inflation coefficients (VIF; 1.07), which were within the suggested threshold [44].

In order to test the interactive relationship of *oversensitivity* and *egocentricity,* an interaction term was calculated using mean centred values in order to minimise multicollinearity arising from entering both the variables and their interaction into the regression equation*:* Interaction = *oversensitivity* (mean-centred) × *egocentricity* (mean-centred). The interaction term was entered in the second block of the hierarchical regression in order to evaluate the contribution of the interaction *separately* from the individual variables. Adding age into the regression analysis did not change the pattern or significance of the results.

With *oversensitivity* and *egocentricity* as independent variables in block 1 and the interaction entered into block 2, the dependent variable for each of the six hierarchical regression analyses was as follows:

1. Time: “On average, how much time do you spend looking at social media each day?”
2. Frequency: “How often do you post to your social media accounts?”
3. Likes/comments: “I care about the number of likes and/or comments I receive on the things I post, and I feel good about myself when this number is high”
4. Jealousy: “I feel upset and/or jealous when I see other people posting positive updates about their life and I find myself comparing my life to theirs”
5. Negative remarks: “One negative remark on my post would make me feel badly and would ruin my day”
6. Portrayal difference: “How I portray myself on social media is different to how I am in real life”

Each regression analysis was cross-validated in order to ascertain whether the estimated regression model is generalisable beyond the sample data used to fit it. We randomly split the sample 70/20 so that 70% of the dataset could act as the model-building set and the remaining 20% could serve as a validation (prediction) set.

#### 2.3.3. ANOVA

A series of one-way ANOVAs were used to assess significant differences in *oversensitivity* and *egocentricity* and social media habits, i.e., time of usage and reason for usage. We also categorised participants based on their *oversensitivity* and *egocentricity* scores. *Oversensitivity* and *egocentricity* scores were averaged and standardised before categorising into three groups based on 1 Standard Deviation *(S.D)* above or below the mean; (1) people with a difference of 1 *S.D.* higher in oversensitivity and lower in egocentricity (2) people with an equal balance of both forms of vulnerable narcissism, and (3) people with a difference of 1 *S.D.* higher egocentricity and lower oversensitivity to evaluate significant differences in social media usage between different vulnerable narcissist phenotypes.

## 3. Results

### 3.1. Descriptive Statistics of Participants

Participants reported their preferred social media, with 36.5% favouring Instagram, 20% Snapchat, 18.3% Twitter, 15.7% Facebook, 6% TikTok, and 3.5% other platforms. Participants also reported their reasons for using social media, with 92% to waste time, 86% to stay in touch with friends and family, 74.6% to keep up to date with news, 49.6% to post videos and pictures of themselves, 31.6% to post updates about their lives, 21.1% buying and selling items, 20.4% to make new friends, 12.3% to connect with professional contacts, 11.4% to vent about difficulties, 9.6% to promote a business or make money, and 7.9% to make a following and become well-known. Age significantly correlated with how much time participants spent looking at social media [*r* = 0.41, *p* < 0.001] but none of the other social media variables or vulnerable narcissism scores (all *p* = 0.08–0.908). Due to the female majority of participants in our sample, we did not perform statistical analyses on gender.

### 3.2. Factor Analysis of HSNS

Data from all participants were entered into the factor analysis. The results of the varimax rotation for the tasks are shown in Table 1. Only factor loadings greater than 0.30 are shown. A two-factor solution was derived based on eigenvalues greater than 1, which cumulatively accounted for 45% of the variance. Data were assessed for the adequacy of factor analytic methods. Bartlett’s test was highly significant [χ^2^_(__45__) =_ 226.08, *p* < 0.001], suggesting that variable correlations did not form an identity matrix. Measures of sampling adequacy were also sufficient (KMO = 0.69). Item one loaded onto two factors but more so on factor 2. Therefore, factor 1 included items two, three, seven, and nine, while items one, four, five, six, eight, nine, and ten loaded onto factor 2. These loadings replicate the factor structures observed by Stone and Bartholomay [38] to represent the Oversensitivity (Factor 1) and Egocentricity (Factor 2) subscales of the HSNS. A confirmatory factor analysis (CFA) revealed a good fit of this model; Comparative Fit Index (CFI) = 0.947; Root Mean Square Error of Approximation (RMSEA) = 0.076. The internal consistency for oversensitivity was acceptable in our sample (α = 0.76). However, internal consistency for egocentricity was moderate (α = 0.59). Overall, internal consistency for the full HSNS was α = 0.70.

### 3.3. Subfactors of Vulnerable Narcissism Which Are Associated with Social Media Use

Using the subscales derived from the factor analysis, we computed two variables: *Oversensitivity* and *Egocentricity*. We entered the subscales into a series of hierarchical regression analyses to evaluate their association with social media use (Table 2). The test of excessive significance revealed a success rate of 0.67, a median observed power of 68% and a deflation (negative inflation rate) of −0.009. The r-index = 0.68 indicates that our findings can be replicated in X * 0.68 follow-up studies.

#### 3.3.1. Time Spent on Social Media Sites

The regression model was significant [F(2, 114) = 5.13, *p* < 0.007, R^2^ = 0.08, Cohen’s *f*^2^ = 0.09] and showed that higher oversensitivity scores was significantly related to more time spent online (β = 0.30, *t* = 3.20, *p* = 0.002). However, time spent on social media sites was not related to egocentricity. Even when adding age into the regression analysis, given that age correlated with time spent online, this did not change the pattern or significance of the results. The interaction of *Oversensitivity* and *Egocentricity* was a significant contributor to the model (β = −0.20, *t* = −2.15, *p* = 0.034, R^2^change = 0.037). However, when entering egocentricity into the first block of the model alone, it was not significantly related to time spent online (β = 0.01, *t* = 0.12, *p* = 0.91), suggesting an indirect contribution of egocentricity.

#### 3.3.2. Frequency of Posts to Social Media

The regression model was significant [F(2, 114) = 3.50, *p* < 0.034, R^2^ = 0.06, Cohen’s *f*^2^ = 0.06] and showed that higher oversensitivity scores significantly related to more frequent posts online (β = 0.24, *t* = 2.56, *p* = 0.012). However, frequency of posts to social media sites was not related to egocentricity. The interaction variable was not a significant contributor to the model (β = 0.12, *t* = 1.25, *p* = 0.21).

#### 3.3.3. Likes and Comments

The regression model was significant [F(2, 114) = 14.06, *p* < 0.001, R^2^ = 0.20, Cohen’s *f*^2^ = 0.25] and showed that higher oversensitivity scores was significantly related to greater concern about obtaining likes and comments (β = 0.46, *t* = 5.30, *p* < 0.001). However, likes and comments were not related to egocentricity. The interaction variable was not a significant contributor to the model (β = 0.13, *t* = 1.49, *p* = 0.14).

#### 3.3.4. Jealousy

The regression model was significant [F(2, 114) = 19.54, *p* < 0.001, R^2^ = 0.26, Cohen’s *f*^2^ = 0.35] and showed that higher oversensitivity scores was significantly related to greater jealousy and social comparison to others (β = 0.50, *t* = 5.91, *p* < 0.001). However, jealousy and social comparison were not related to egocentricity. The interaction variable was not a significant contributor to the model (β = −0.11, *t* = −1.30, *p* = 0.20).

#### 3.3.5. Negative Remarks

The regression model was significant [F(2, 114) = 31.89, *p* < 0.001, R^2^ = 0.36, Cohen’s *f*^2^ = 0.54] and showed that higher oversensitivity scores was significantly related to greater concern about negative remarks (β = 0.62, *t* = 7.98, *p* < 0.001). Conversely, greater egocentricity scores were significantly related to less concern about one negative remark (β = −0.18, *t* = −2.34, *p* = 0.021). The interaction variable was not a significant contributor to the model (β = 0.06, *t* = 0.76, *p* = 0.45). When entering egocentricity into the first block of the model alone, it was not related to concerns over negative remarks (β = 0.025, *t* = 0.27, *p* = 0.79), only becoming significant when oversensitivity was entered, suggesting that the relationship with egocentricity is moderated by oversensitivity. Oversensitivity remained significant regardless of inclusion of egocentricity and the interaction term.

#### 3.3.6. Portrayal Difference

The regression model was significant [F(2, 114) = 13.42, *p* < 0.001, R^2^ = 0.19, Cohen’s *f*^2^ = 0.24] and showed that both higher oversensitivity (β = 0.35, *t* = 3.93, *p* < 0.001) and higher egocentricity (β = 0.20, *t* = 2.27, *p* = 0.025) scores were significantly related to a greater difference in how they portray themselves on social media compared to real life. The interaction variable was not a significant contributor to the model (β = −0.10, *t* = −1.20, *p* = 0.24). When entering egocentricity into the first block of the model alone, it was independently related to portrayal difference (β = 0.29, *t* = 3.18, *p* = 0.002), suggesting that the relationship with egocentricity is independent of oversensitivity.

#### 3.3.7. Cross-Validation

Five out of the six regression analysis were successfully cross-validated. Regression analyses for time spent online, caring about likes and comments, feeling jealousy and a need for social comparison, and caring about negative remarks were unchanged after cross-validation on the 30% sample. However, for frequency of posting online, oversensitivity did not survive cross-validation and was no longer significant (*p* = 0.17).

### 3.4. Social Media Usage

#### 3.4.1. Time of Usage

Participants were asked when they check their social media. Using a series of one-way ANOVAs, we found that participants who check their phone when they wake up [F(1, 113) = 6.07, *p* = 0.015, η_p_^2^ = 0.05], before they go to bed [F(1, 113) = 5.15, *p* = 0.025, η_p_^2^ = 0.04] and in the bathroom [F(1, 113) = 5.24, *p* = 0.024, η_p_^2^ = 0.05] had significantly higher oversensitivity but not egocentricity scores. However, participants who check their phone during meal times had significantly higher egocentricity scores [F(1, 113) = 7.36, *p* = 0.008, η_p_^2^ = 0.06]. Checking social media when in social situations was associated with both higher oversensitivity [F(1, 113) = 7.14, *p* = 0.009] and egocentricity [F(1, 113) = 6.27, *p* = 0.014, η_p_^2^ = 0.06] scores.

#### 3.4.2. Reason for Usage

Participants were asked why they used social media. Using a series of one-way ANOVAs, we found that participants who use social media to post images and videos of themselves [F(1, 113) = 5.42, *p* = 0.022, η_p_^2^ = 0.05] and post updates on their life [F(1, 113) = 6.26, *p* = 0.014, η_p_^2^ = 0.05] had significantly higher oversensitivity but not egocentricity scores [F(1, 113) = 1.21, *p* = 0.27, η_p_^2^ = 0.01; F(1, 113) = 1.76, *p* = 0.19, η_p_^2^ = 0.01].

#### 3.4.3. Vulnerable Narcissism Phenotypes

**Figure 1:**
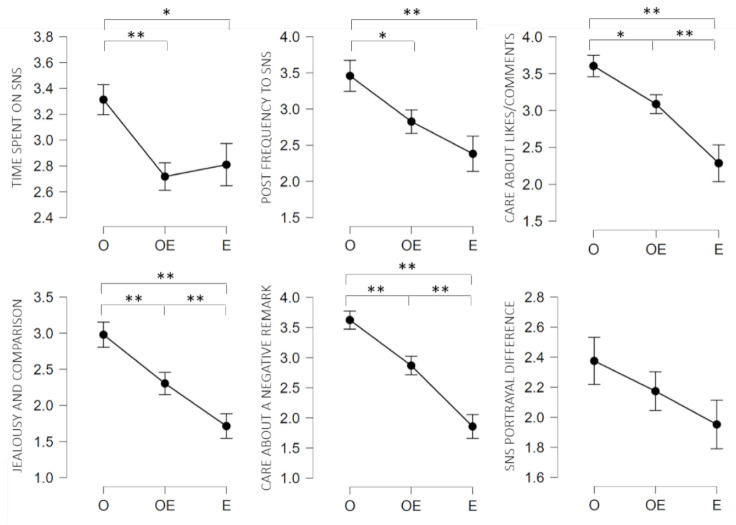
Charts to show social media response separated by Oversensitive (O), equal (OE), and Egocentric (E) phenotypes of vulnerable narcissism. Error bars represent standard error of the mean; * *p* < 0.05, ** *p* < 0.01.

We classified participants into three subgroups (see statistical analysis section for criteria): (1) O—people higher in oversensitivity and lower in egocentrism (*n* = 48); (2) EO—people with an equal balance of both forms of vulnerable narcissism (*n* = 46); and (3) E—people with higher egocentricity and lower oversensitivity (*n* = 21). A series of ANOVAs revealed a main effect of phenotype in five of the six social media variables, i.e., time spent on social media [F(2, 112) = 7.85, *p* < 0.001, η_p_^2^ = 0.12], frequency of posts to social media [F(2, 112) = 5.96, *p* = 0.003, η_p_^2^ = 0.10], caring about likes and comments [F(2, 112) = 13.43, *p* < 0.001, η_p_^2^ = 0.19], jealousy and social comparison [F(2, 112) = 10.98, *p* < 0.001, η_p_^2^ = 0.16], and a negative remark ruining their day [F(2, 112) = 22.47, *p* = 0.001, η_p_^2^ = 0.29], but not portraying themselves differently on social media [F(2, 112) = 1.53, *p* = 0.221, η_p_^2^ = 0.03]. Post Hoc comparisons with adjusted *p* values for multiple comparisons revealed that oversensitive phenotypes spent more time online, posted more frequently, cared more about likes and comments, felt more jealousy and a need for social comparison, and one negative remark was more likely to ruin their day as compared to egocentric phenotypes (see Figure 1).

## 4. Discussion

The present study aimed to investigate whether vulnerable narcissism was associated with aspects of social media use. Our exploratory factor analysis suggested a two-factor model of vulnerable narcissism with the distribution of item loadings replicating recent findings by Stone and Bartholomay [38]. A confirmatory analysis also suggested that this two-factor model was a good fit for the data. These findings further support a two-factor model of vulnerable narcissism, namely *oversensitivity* and *egocentricity*, which were utilised to assess the differential contributions to specific aspects of social media use. We observed that greater oversensitivity was significantly associated with higher scores in all six aspects of social media use. Specifically, time spent on social media, frequency of posts to social media, concerns about numbers of likes and comments, being overly sensitive about negative remarks, feelings of jealousy about other peoples’ social media posts, and a greater difference in how they portray themselves on social media compared to real life. In contrast, we found that greater egocentricity was significantly associated with less concern over negative remarks. Similarly to oversensitivity, we found that higher egocentricity scores was also associated with a greater difference in how they portray themselves on social media compared to real life. In addition, by subgrouping participants into different phenotypes of vulnerable narcissism, we were able to show that people who scored higher in oversensitivity compared to egocentrism showed higher scores for five out of the six social media measures, with the exception of portrayal difference, which was also shown to be related to both forms of vulnerable narcissism in the regression analyses. People who presented with greater egocentricity scores compared to oversensitivity showed a significant reduction in caring about likes, feeling jealous, or concerns over one negative remark as compared to participants who presented with equal levels of both factors of vulnerable narcissism.

### 4.1. Vulnerable Narcissism and Social Media Usage

To date, research that has investigated the links between narcissism and social media use has largely focused on grandiose narcissism, whilst the relationship between social media use and vulnerable narcissism is still relatively unknown. Our results suggest that levels of vulnerable narcissism are related to time spent on social media and frequency of posting to social media. However, this is related more to oversensitivity as opposed to the egocentricity component of vulnerable narcissism. This may be crucial to better understanding the mixed results and variation in previous studies and meta-analyses investigating vulnerable narcissism and social media use [13,23]. Our results indicate that oversensitivity to judgement and preoccupation with what others’ think drives a greater frequency of social media usage rather than the more egocentric self-absorption and self-centeredness trait of vulnerable narcissism. Indeed, vulnerable narcissists often experience high levels of anxiety in social interactions, and therefore, they avoid social contacts in the offline world in favour of social networking sites [45], where they can carefully plan and control self-presentation [29]. However, this may contribute to further excessive immersion into the online world and to the development of problematic social media use that can negatively impact well-being [46]. Indeed, we also observed that people who scored higher in oversensitivity were more likely to use social media as soon as they wake up, as soon as they go to bed, and when they are in the bathroom. Egocentricity, on the other hand, was linked to checking their phone during meal times and both factors of vulnerable narcissism were linked with checking social media when in social situations, i.e., when in the physical company of other people. Spending more time on social media, especially during social situations, further reduces opportunities to make real-world connections and friendships. Recent findings suggests that phubbing (phone snubbing), which refers to a set of behaviours where phone users focus on their mobile phone instead of interacting with their physically proximal companions, is thought to be more prevalent in people with higher vulnerable narcissistic traits [47].

### 4.2. Oversensitivity, Egocentricity, and Social Media

Previous literature has suggested that by engaging in more frequent use of SNSs, there is more opportunity for positive feedback, including positive comments and “likes”, which fosters self-esteem and increases feelings of admiration [13]. This is particularly important for vulnerable narcissists who lack opportunities in the real world due to anxiety and insecurities [46]. Our findings suggest that oversensitivity but not egocentricity is significantly associated with concerns about likes and comments on social media. Furthermore, we observed that people who use SNS to post images, videos, and updates on their life had higher oversensitivity but not egocentricity scores. Finally, when separating participants into subgroups, we observed that those who display more oversensitivity were more likely to care about likes and comments compared to people who show a balance of oversensitivity and egocentricity scores, which was even less for people who displayed predominately higher egocentricity scores. Together, this suggests that vulnerable narcissism is related to caring about likes and comments and is specifically driven by oversensitivity to feedback from others.

We also observed that oversensitivity but not egocentricity was significantly associated with feelings of jealousy over other people’s social media posts. Envy has been historically linked with the grandiose form of narcissism; however, accumulating evidence suggests that envy is driven more by narcissistic vulnerability, not grandiosity [48]. Indeed, vulnerable narcissists may be dispositionally envy-prone [49], especially considering that their behaviours are often driven by feelings of inadequacy [5]. Our findings further suggest that jealousy felt from observing others’ social media posts is specifically related to oversensitivity.

Oversensitivity was also positively associated with concerns over negative remarks on social media posts, whereas egocentricity was negatively associated with concerns about negative remarks. This suggests that the egocentricity aspect of vulnerable narcissism may have protective properties to reduce oversensitivity to negative feedback. However, our results showed that any effect of egocentricity was moderated by oversensitivity. The lack of concern over negative feedback is reflective of more grandiose narcissism. This may lend support to Stone and Bartholomay’s [38] conclusion, suggesting that the oversensitivity factor of the HSNS may be a purer measure of vulnerable narcissism, whereas egocentricity may be more closely aligned with grandiose narcissism. Furthermore, Atlas and Them [50] found that those who score high in grandiose narcissism tend to be less sensitive to criticism, whereas those who score high in vulnerable narcissism tended to be more sensitive to criticism. Nevertheless, our study did not measure grandiose narcissism and is, therefore, not able to assess the relationship between vulnerable *egocentricity* and grandiose narcissism.

We observed that both oversensitivity and egocentricity were significantly related to how different people portray themselves on social media. This is in line with a recent study by Grieve et al. [36], who found that there was a greater difference between personas in vulnerable narcissists as compared to grandiose narcissists. Vulnerable narcissists’ low self-esteem and preoccupation with how others perceive them are likely to drive them to enhance and curate an image online to receive attention and approval. This aligns with the Social Online Self-Regulation Theory, whereby different motivations to use social media all fall within the broader term of self-regulation as the primary goal [25].

## 5. Limitations

There are several limitations of the present study which may have affected the reliability and validity of the results. Firstly, this study used a relatively small sample size of 115 participants, using an opportunistic sample of participants recruited largely from a university population, with the majority of participants aged between 18 to 21 years old. Previous research has indicated that younger people tend to display more narcissistic traits than older adults and are also more likely to engage in social media [51]; therefore, further research will need to ascertain the reliability of these findings in a more age-diverse sample to minimise any mediating effect of age. Furthermore, our sample was largely female, and therefore, caution should be taken in generalising these results across males and females. Greater sample sizes of both males and females would allow for further exploration of sex differences. A larger sample size would also have allowed for a more sophisticated statistical analysis, such as structural equation modelling (SEM). This study also relied solely on self-report measures; therefore, future research should make use of more objective measures for social media use, such as taking data directly from participants’ social media accounts. Finally, the egocentricity factor showed a less than acceptable internal consistency in comparison to Stone and Bartholomay [38]; therefore, further evidence is needed to ascertain the item loadings and their potential predictive value for assessing social media use.

## 6. Conclusions

Social media provides an ideal platform for people with narcissistic traits to promote and to satisfy a need for attention and admiration. This is especially relevant for people who display vulnerable narcissistic traits, where social interactions in the real world are problematic. The present study demonstrated that there is a significant relationship between vulnerable narcissism and social media use, particularly in relation to *oversensitivity*. These findings raise concerns about the utility of the HSNS as a pure measure of vulnerable narcissism and highlights a need to reconsider this scale as multidimensional, tapping into the core of vulnerable narcissism (*oversensitivity*), as well as aspects of grandiose narcissism (*egocentricity*). It is also important to identify and monitor individuals high in oversensitivity to prevent the onset of problematic social media use, which has the potential to further oversensitise these individuals to feedback from others. Adolescents, in particular, have been shown to have heightened sensitivity to social evaluation, reflected by increased recruitment of socio-affective brain circuitry, which may make them particularly vulnerable to heightened mood and anxiety disorders throughout the life span (for a review, see Somerville, 2013 [52]). Given that social media is more popular than ever, and is likely to remain a permanent and integral part of society for the purposes of communication, learning, and entertainment, future research should explore how existing and future social media platforms can be improved to protect the wellbeing of individuals, particularly those who are highly sensitive. For instance, removing the number of likes shown on photos and posts online may reduce individuals’ concerns about feedback, reduce feelings of jealousy and the need for social comparison, and reduce the disparity of their online portrayal. It is also important for parents and educational settings to play a role in reducing the risk of problematic social media use and protecting the wellbeing of individuals who are particularly oversensitive. For instance, offering free counselling sessions could help to build self-esteem and reduce the need for online approval, and educating young people on the nature of social media could help individuals to become aware of the altered images and curated lifestyles that are often posted online. Indeed, adolescent’s perceptions of supportive and warm relationships with parents and educators lay a foundation for adolescent wellbeing [53] and emotional reactivity to peer evaluation [54]. Therefore, it is important to identify those who display high levels of oversensitivity in order to offer strategies and interventions which may attenuate the detrimental effects of social media and make social media a more positive and healthy experience for individuals who find it difficult to separate their feelings of self-worth and self-confidence from the unrealities of the online world.

## Figures and Tables

**Table 1:** Summary of HSNS factor loadings for oversensitivity and egocentricity.

Items	Oversensitivity	Egocentricity
(1) I can become entirely absorbed in thinking about my personal affairs, my health, my cares, or my relations to others.	0.335	0.348 *
(2) My feelings are easily hurt by ridicule or the slighting remarks of others.	0.786 *	−0.137
(3) When I enter a room, I often become self-conscious and feel that the eyes of others are upon me.	0.739 *	0.115
(4) I dislike sharing the credit of an achievement with others.	−0.108	0.548 *
(5) I feel that I have enough on my hands without worrying about other people’s troubles.	−0.078	0.652 *
(6) I feel that I am temperamentally different from most people.	0.378	0.539 *
(7) I often interpret the remarks of others in a personal way.	0.839 *	0.044
(8) I easily become wrapped up in my own interests and forget the existence of others.	0.154	0.581 *
(9) I dislike being with a group unless I know that I am appreciated by at least one of those present.	0.635 *	0.121
(10) I am secretly “put out” or annoyed when other people come to me with their troubles, asking me for my time and sympathy.	0.096	0.638 *

* indicates which of the two factors each of items loads on to.

**Table 2:** Summary of multiple regression analyses for oversensitivity and egocentricity scores in relation to social media variables.

	Independent Variables					
	Oversensitivity				Egocentricity	
Dependent Variables	β	t	*p*	β	t	*p*
Time online	0.30	3.20	0.002 **	−0.06	−0.69	0.492
Post frequency	0.24	2.56	0.012 *	0.12	1.29	0.200
Likes/comments	0.46	5.30	<0.001 **	0.14	1.57	0.122
Jealousy	0.50	5.91	<0.001 **	0.04	0.47	0.636
Negative remarks	0.62	7.98	<0.001 **	−0.18	−2.34	0.021 *
Portrayal difference	0.35	3.93	<0.001 **	0.20	2.27	0.025 *

* *p* < 0.05, ** *p* < 0.01.

## Data Availability

Data is available upon request to the authors.
